# Human Factors and Organizational Issues: Contributions from 2023

**DOI:** 10.1055/s-0044-1800745

**Published:** 2025-04-08

**Authors:** Anthony Solomonides, Yalini Senathirajah

**Affiliations:** 1 Research Institute, Endeavor Health, Evanston, IL USA; 2 University of Pittsburgh, Pittsburgh, PA USA

**Keywords:** International Medical Informatics Association Yearbook, Human Factors, Organizational issues, Digital Health, Usability

## Abstract

**Objectives** : To review publications in the field of Human Factors and Organisational Issues (HF&OI) in the year 2023 and to assess major contributions to the subject.

**Methods** : A bibliographic search was conducted following further refinement of standardized queries used in previous years. Sources used were PubMed, Web of Science, and referral via references from other papers. The search was carried out in February 2024, and (using the PubMed article type inclusion functionality) included clinical trials, meta-analyses, randomized controlled trials, reviews, case reports, classical articles, clinical studies, observational studies, comparative studies, and pragmatic clinical trials.

**Results** : Among the 513 returned papers published in 2023 in the various areas of HF&OI, 87 were identified for full review that resulted in a shortlist of 12 finalists and finally three best papers from among these. As in previous years, topics showed development including increased use of Artificial Intelligence (AI) and digital health tools, advancement of methodological frameworks for implementation and evaluation as well as design, and trials of specific digital tools.

**Conclusions** : Recent literature in HF&OI continues to focus on both theoretical advances and practical deployment, with focus on areas of patient-facing digital health, methods for design and evaluation, and attention to implementation barriers.

## 1. Introduction

We begin this edition, as we have done in earlier reviews, with an acknowledgement of the difficulties presented by as broad and diverse a subject as Human Factors and Organizational Issues (HFOI). It touches on a multiplicity of relevant domains in informatics and varies in its adopted methods from the study of physical devices and electronic platforms through clinical trials of novel interventions to the new sciences of implementation and teamwork. Amid all this wealth of material, we will no doubt have missed or been unaware of some excellent contribution, possibly published in a specialist domain journal. We hope this admission explains any egregious omission.

These observations are all the more significant when we come to the selection of “best papers”—in quotation marks to signal the limitations of our collective process. Between the present authors, the editors of the Yearbook and the half dozen or so reviewers, at most ten individuals have been involved in this selection: we cannot claim to be representative of the HFOI community. A focal feature for our section is the review of HFOI by Drs. Andre Kushniruk and David Kaufmann, who capture the dynamism and vibrancy of the field with the sure touch of scientists discussing their own field. They view innovations and gaps in their discipline at the micro, meso, and macro levels, and introduce a number of themes we have not previously reviewed under HFOI, not least the explosive growth in—often uncritical—AI adoption. Their analysis offers an excellent overview.

## 2. Methods

**Figure 1.**
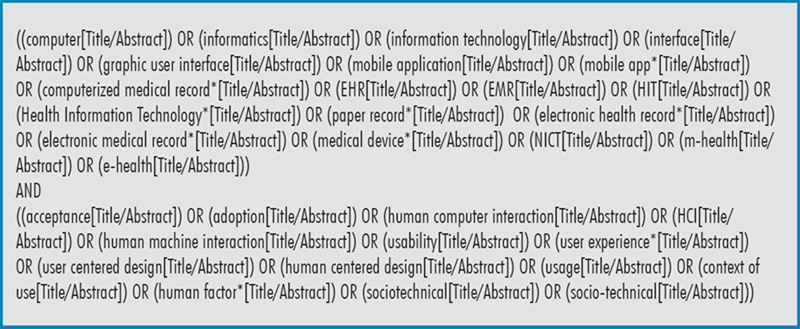
Search strategy queries for HFOI.

**Figure 2.**
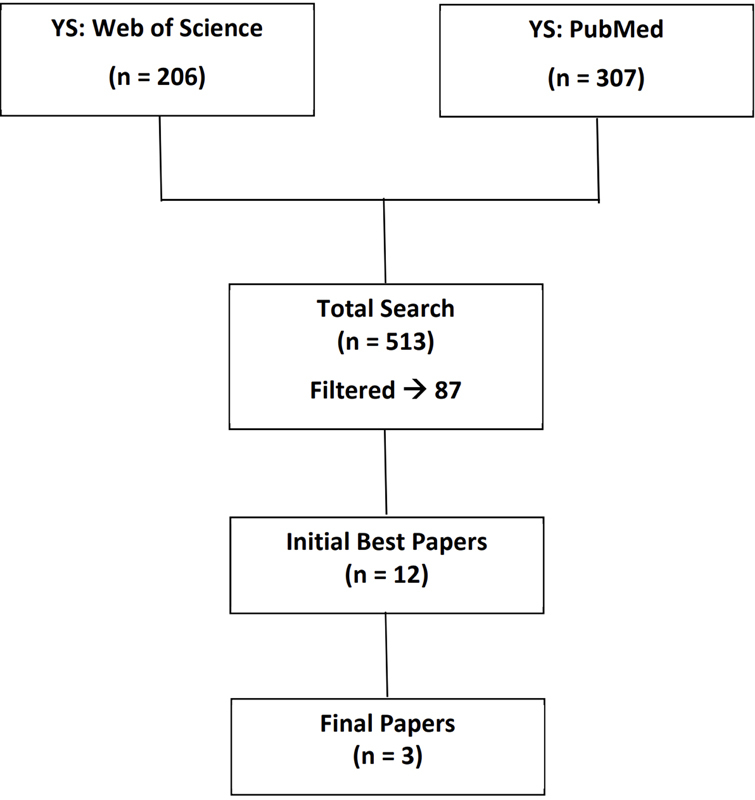
Search strategy queries for HFOI.

The search strategy adopted in the last two years yielded an almost unmanageable number of contributions. For 2023 we amended the query (see Figure 1 ) on PubMed & Web of Science® resulting in 513 papers which we filtered down to 87 based on relevance through independent screening of titles and abstracts by the two section editors. The two editors made individual selections and through a process of reconciliation agreed on a selection of 12 candidate best papers. Five additional reviewers reduced the list to five from which the authors selected the top three (see Figure 2 and Appendix).

## 3. Results

We noted a bias towards soft and qualitative methods in the papers identified by our search strategy. We were not able to locate this bias in our query compared to earlier years, but this bias persists in the selection of papers we discuss below in this section.

### 3.1 Wellbeing and Physical Health

The value of physical exercise both for physical and for mental health is well established, but finding the means to motivate individuals to engage, especially elderly patients, presents a significant challenge. Among community-dwelling elderly, the risk of a fall with injury provides both an impetus to exercise and a reason to avoid it. This active research domain is represented here by two studies, one by Ambrens *et al.* [ 1 ] in Australia, and the other by Shear *et al.* [ 2 ] in the US. In-person physical training to improve balance works, but it is not scalable. In the first of these studies, older adults who engaged in the program enjoyed the flexibility of the online delivery of StandingTall, an app under development, but offered extensive criticism of the design of the program, including a perception of ageism seemingly implicit in the application. In the US-based study, the team developed its own app, ASPIRE, with scalability in mind. The app scored well on the System Usability Scale and interfaced successfully with at least two EHR platforms. The contrast between these two promising approaches lies primarily in the decision to develop rather than adopt a solution. Another Australian study [ 3 ] explored the use of Fitbit devices in the Active for Life computer-based program, again aimed at older adults. Notwithstanding mixed results, all these studies found their trial supported the virtual approach to exercise. A group of patients who are strongly recommended to remain active mentally and physically are those with a diagnosis of Parkinson's. A study from the UK [ 4 ] adopted combined visual and cognitive (“visuo-cognitive”) training program to facilitate home-based rehabilitation for people with Parkinson's. Acceptance of the technology and its usability were significant determinants of success. Adaptation of the method to the individual patient was necessary for optimal results.

An entirely different time when novel modes of support may be valued is the postpartum and breastfeeding period for mothers. A study from Spain [ 5 ] explores the use of a mobile app, LactApp®, on sustained breastfeeding in the first six months. The app-supported approach led to about the same results at six months as the standard of care, but was superior at 15 days, i.e., better at initiation and early weeks of breastfeeding. Postpartum care for low-income mothers presents an altogether different problem. A study at several academic medical centers in the US [ 6 ] demonstrated the effectiveness of navigators through an analysis of their logs of contacts with patients, care teams, and community organizations. Navigation proved especially valuable in the first three months.

### 3.2 Mental and Behavioral Health

A paper from the Netherlands [ 7 ] links the stress of poor vision to fatigue and introduces a computer-based behavioral therapy application, E-nergEYEze, that performs promisingly in a soft launch and then a feasibility study. A full clinical trial should be the next step. Another group that has experienced burdensome stress is the health professionals working through the COVID-19 pandemic. A South African study [ 8 ] explored the use of a mental health app to help healthcare workers to deal with such stress and reports significant reduction in anxiety and acute stress disorder severity. Young adult cancer survivors suffer stressful symptoms, with pain, fatigue, and distress ranked at the top of the list. A therapeutic intervention [ 9 ] coupled with a mobile application led to the development of a behavioral symptom management program.

Lifestyle interventions are well represented in our selection. Healthy diet support is an active topic, illustrated here by a contribution from India [ 10 ] that adopted a multichannel approach, combining SMS, WhatsApp, and a “SMART Eating” kit distributed in person to reduce fat, sugar and salt intake while enhancing fruits and vegetables. A Norwegian study [ 11 ] developed and tested an app aimed at lifestyle change towards healthier behaviors for people at risk of Type II diabetes. A group in Amsterdam conducted a useful systematic review and meta-analysis of conversational agents (CAs) to support smoking cessation. [ 12 ] Overall, their findings support CAs, but they also found variable quality and evidence of publication bias.

A study from Germany [ 13 ] reports an approach to empathy development through mindfulness, including training delivered on mobile apps. Empathy towards self and towards others improved, although the effects were relatively small. Finally, a group from Manchester, UK, report on Actissist, [ 14 ] a co-produced digital health intervention to support people with early psychosis, where it is known that engagement is critical.

### 3.3 Technology Acceptance, Economics, EHRs

What is the consumer's point of view of the physical and mental wellbeing apps and platforms we have discussed? A major framing of this question is through models of technology acceptance (TA). A thorough meta-analysis from Brazil [ 15 ] adopts a widely used TA model (UTAUT2 [ 16 ]) to identify factors that contribute to a behavioral intention to use mobile health technologies among consumers. Acceptance of new technologies to professionals is also an area of intense interest. Among the most successful AI applications in healthcare have focused on imaging. A scoping review [ 17 ] from Sydney, Australia, explores the acceptability of imaging-related AI to professionals. The authors identify a wide range of factors that impinge on this and find that many of the studies their PRISMA-inspired search simply ignore many of these factors, but also observe that in the case of AI human-centeredness is a critical element in the acceptance of the technology. The economics of digital interventions in healthcare come under scrutiny in a systematic review from Singapore, Germany, and Canada [ 18 ]. Noting the accelerated introduction of digital platforms to support remote care through the COVID-19 pandemic, the authors consider the economic impact of this development. Of 18 studies included in their review, the authors note that in 12 the digital intervention was shown to be cost effective, in five it proved cost saving, while in the final one it showed limited cost effectiveness.

At the more traditional end of the technology spectrum, two interesting studies explore the completeness and usefulness of problem lists in EHRs. In [ 19 ] a team from the US reviews the use of a computer decision support intervention to enhance problem list completeness. They find that while problem lists improve, outcomes largely remain the same. Somewhat contrasting, a Dutch team report from Amsterdam [ 20 ] that curated problem lists, essentially meaning discretely documented and annotated diagnoses, led to better and more rapid decision making in medication prescription decisions.

### 3.4 Physical Recovery, Wearable and Implantable Devices

Rehabilitation following an acute event or injury is an area ripe for e-health, given the scarcity and cost of human physiotherapists and trainers. A case in point is recovery from stroke. A team from China report [ 21 ] on a project that eschews expensive devices but opts for simple wearables and computer interaction and compares outcomes with occupational therapy. The results are sufficiently encouraging for a future deployment with virtually no human mediation. Recovery from abdominal surgery, even if minimally invasive, takes time and requires support. A Dutch study [ 22 ] compares standard of care plus a “placebo website”—one offering only advice—with an intervention including a website and mobile app with an e-consult function and an activity tracker. In summary, the intervention allowed participants to return to normal activities nearly two weeks earlier than those in the control group.

In sharp contrast, we conclude with two studies that sought to impact the functioning of the recovering brain directly and thus to enhance a brain-computer interface (BCI). Both are premised on the power of motor imaging, a mental rehearsal of motion, to exploit neuroplasticity in restoring function to a damaged brain. A study from Rome, Italy, [ 23 ] proposes to explore the use of an electroencephalography (EEG)-based BCI to improve motor imaging practice. This clinical trial was registered in 2020 and is still recruiting. The criteria for its statistical analysis shed light on the complexity of the problem under consideration. Concurrently, a team from Xi'an, China, reports [ 24 ] on a different motor imagery training intervention which exploits errors through an associated potential. This approach led to a system that recognizes the user's intention correctly in > 80% of cases. Certainly, the impression imparted by these two studies is that functional consumer BCIs cannot be long in coming.

## 4. Conclusion

In this synopsis, we have discussed 23 papers selected by the two authors; we discuss the three best papers listed in Table 1 in the Appendix. All reviewers placed these in the top scores. The papers cover topics with interesting and important methods and conclusions. A content summary and brief discussion of the three best papers can be found in the Appendix.

**Table 1.** Best papers on HF&OI 2023 listed by first author's name.

• Ruissen MM, Torres-Peña JD, Uitbeijerse BS, Arenas de Larriva AP, Huisman SD, Namli T, Salzsieder E, Vogt L, Ploessnig M, van der Putte B, Merle A, Serra G, Rodríguez G, de Graaf AA, de Koning EJP, Delgado-Lista J, Sont JK; POWER2DM Consortium. Clinical impact of an integrated e-health system for diabetes self-management support and shared decision making (POWER2DM): a randomised controlled trial. Diabetologia. 2023 Dec;66(12):2213-2225. doi: 10.1007/s00125-023-06006-2. • Teo SH, Chew EAL, Ng DWL, Tang WE, Koh GCH, Teo VHY. Implementation and use of technology-enabled blood pressure monitoring and teleconsultation in Singapore's primary care: a qualitative evaluation using the socio-technical systems approach. BMC Prim Care. 2023 Mar 16;24(1):71. doi: 10.1186/s12875-023-02014-8. • Yao Y, Dunn Lopez K, Bjarnadottir RI, Macieira TGR, Dos Santos FC, Madandola OO, Cho H, Priola KJB, Wolf J, Wilkie DJ, Keenan G. Examining Care Planning Efficiency and Clinical Decision Support Adoption in a System Tailoring to Nurses' Graph Literacy: National, Web-Based Randomized Controlled Trial. J Med Internet Res. 2023 Aug 11;25:e45043. doi: 10.2196/45043. PMID: 37566456; PMCID: PMC10457701.
